# Homocysteine in Schizophrenia: Independent Pathogenetic Factor with Prooxidant Activity or Integral Marker of Other Biochemical Disturbances?

**DOI:** 10.1155/2021/7721760

**Published:** 2021-10-13

**Authors:** T. V. Zhilyaeva, A. S. Piatoikina, A. P. Bavrina, O. V. Kostina, E. S. Zhukova, T. G. Shcherbatyuk, A. S. Blagonravova, E. E. Dubinina, G. E. Mazo

**Affiliations:** ^1^Privolzhsky Research Medical University, Russia; ^2^Nizhny Novgorod Clinical Psychiatric Hospital No. 1, Russia; ^3^Nizhny Novgorod Research Institute for Hygiene and Occupational Pathology, Russia; ^4^Pushchino State Institute of Natural Science, Russia; ^5^Moscow Region State University, Russia; ^6^V.M. Bekhterev National Medical Research Center for Psychiatry and Neurology, Russia

## Abstract

A wide range of studies have demonstrated that hyperhomocysteinemia is associated with the risk of schizophrenia, but currently available assumptions about the direct involvement of homocysteine (Hcy) in the pathogenesis of schizophrenia are hypothetical. It is possible that in vivo Hcy is only a marker of folate metabolism disturbances (which are involved in methylation processes) and is not a pathogenetic factor per se. Only one study has been conducted in which associations of hyperhomocysteinemia with oxidative stress in schizophrenia (oxidative damage to protein and lipids) have been found, and it has been suggested that the oxidative stress may be induced by the elevated Hcy in schizophrenic patients. But the authors did not study the level of reduced glutathione (GSH), as well as possible causes of hyperhomocysteinemia—disturbances of folate metabolism. The aim of this work is to analyze the association of Hcy levels with the following: (1) redox markers in schizophrenia GSH, markers of oxidative damage of proteins and lipids, and the activity of antioxidant enzymes in blood serum; (2) with the level of folate and cobalamin (В12); and (3) with clinical features of schizophrenia measured using the Positive and Negative Syndrome Scale (PANSS). 50 patients with schizophrenia and 36 healthy volunteers, matched by sex and age, were examined. Hcy in patients is higher than in healthy subjects (*p* = 0.0041), and this may be due to the lower folate level in patients (*p* = 0.0072). In patients, negative correlation was found between the level of Hcy both with the level of folate (*ρ* = −0.38, *p* = 0.0063) and with the level of B12 (*ρ* = −0.36, *p* = 0.0082). At the same time, patients showed higher levels of oxidative modification of serum proteins (*p* = 0.00046) and lower catalase (CAT) activity (*p* = 0.014). However, Hcy is not associated with the studied markers of oxidative stress in patients. In the group of patients with an increased level of Hcy (>10 *μ*mol/l, *n* = 42) compared with other patients (*n* = 8), some negative symptoms (PANSS) were statistically significantly more pronounced: difficulty in abstract thinking (N5, *p* = 0.019), lack of spontaneity and flow in conversation (N6, *p* = 0.022), stereotyped thinking (N7, *p* = 0.013), and motor retardation (G7, *p* = 0.050). Thus, in patients with schizophrenia, hyperhomocysteinemia caused by deficiency of folate and B12 is confirmed and can be considered a marker of disturbances of vitamin metabolism. The redox imbalance is probably not directly related to hyperhomocysteinemia and is hypothetically caused by other pathological processes or by an indirect effect of Hcy, for example, on the enzymatic antioxidant defence system (CAT activity), which requires further exploration. Further study of the role of Hcy in the pathogenesis of schizophrenia is relevant, since the proportion of patients with hyperhomocysteinemia is high and correlations of its level with negative symptoms of schizophrenia are noted.

## 1. Introduction

Schizophrenia is a chronic disabling multifactorial psychiatric disorder with a broad spectrum of clinical manifestations. Due to the lack of knowledge on its pathogenesis, the selection of effective treatments for schizophrenia remains challenging, especially for negative and cognitive symptoms, which influence patients' everyday functioning. In this regard, the search for biomarkers amenable to correction, potentially involved in pathogenesis, is of particular relevance.

A wide range of studies have demonstrated that hyperhomocysteinemia is associated with the risk of schizophrenia regardless of the age and sex of patients [1–5]. The currently available assumptions about the direct involvement of homocysteine (Hcy) in the pathogenesis of schizophrenia are hypothetical, based on experiments with animals [6–8] or cell culture [9], in which Hcy causes oxidative damage to neuronal tissue and vascular intima. In these studies, extremely high levels of Hcy were artificially created.

Hcy is a sulfhydryl-containing amino acid, an intermediate product in the normal biosynthesis of the amino acids methionine and cysteine. Hcy is produced via demethylation of dietary methionine, which undergoes S-adenosylation and forms S-adenosylmethionine (SAM, the principal methyl donor for all methylation reactions in cells). The demethyation reaction leads to the formation of SAH (S-adenosylhomocysteine). Hydrolysis of SAH leads to the formation of Hcy and adenosine. In case of methionine deficiency, Hcy can be remethylated to form methionine with the participation of folates and cobalamin (В12) [10]. In the case of folate and В12 deficiency, Hcy remethylation to methionine is impaired and Hcy accumulates in excess amounts (causing hyperhomocysteinemia). So, it is possible that *in vivo* Hcy is only a marker of folate metabolism disturbances (which are involved in methylation processes [11], including methylation of catecholamines [12]), and is not a pathogenetic factor per se.

Another way of Hcy utilization is the transsulfuration pathway, the synthesis of reduced glutathione (GSH), which is the most important antioxidant of neuronal tissue [13], as well as taurine, which can promote neurogenesis in the hippocampus [14]. GSH is the main nonenzymatic antioxidant that protects cells from the toxic effect of reactive oxygen and nitrogen species by binding and eliminating them. GSH deficiency in schizophrenia has been established in numerous studies [15, 16]. GSH depletion in the brain is a common finding in patients with neurodegenerative diseases and can cause neurodegeneration prior to disease onset [17]. Therefore, the *in vivo* potential pathogenetic effect of Hcy can be compensated by donating GSH. Moreover, it is possible that in the complexity of pathobiochemical disturbances in the central nervous system hyperhomocysteinemia can play a protective role due to the described mechanism.

So far, only one small (*n* = 19) study has been conducted in which associations of hyperhomocysteinemia with oxidative stress in schizophrenia (oxidative damage to proteins and lipids) have been found and it has been suggested that oxidative stress may be induced by the elevated Hcy in patients [18]. However, the authors did not study the level of GSH, as well as possible causes of hyperhomocysteinemia—disturbances of folate metabolism. Therefore, the opposite causation is not excluded: an increase in Hcy levels due to oxidative stress, in particular, due to blockade of the methylation pathway—a compensatory reaction aimed at increasing the synthesis of GSH, which was previously described in experimental studies on biochemistry without regard to schizophrenia [19].

The aim of this work is to analyze the association of Hcy levels with the following: (1) with redox markers of schizophrenia GSH, markers of oxidative damage of proteins and lipids, and the activity of antioxidant enzymes in blood serum; (2) with the level of folate and cobalamin; and (3) with clinical features of schizophrenia measured using the Positive and Negative Syndrome Scale (PANSS).

## 2. Methods

50 patients with schizophrenia and 36 healthy volunteers, matched by sex and age, were examined. The criteria for inclusion of patients in the study were confirmation of the diagnosis of schizophrenia using the Mini International Neuropsychiatric Interview for Diagnostic and Statistical Manual, Version 5 (M.I.N.I. for DSM-5) and patient's ability to give informed consent to participate in the study. The criteria for inclusion of healthy volunteers in the study were the following: the absence of psychiatric disorders registered in their medical history (a healthy volunteer is not observed by a psychiatrist and has not previously consulted by psychiatrists about mental health problems), social maladjustment, and drug abuse; compliance of sex and age with the patients are included in the study. The criteria for inclusion of all participants (both patients and healthy volunteers) in the study were the absence of chronic somatic diseases and neurological disorders associated with oxidative stress (bronchial asthma, infectious, and other immune-mediated inflammatory rheumatic disorders, oncological disorders, genetic, chromosomal, hereditary diseases) and absence of intake of any antioxidants and vitamins, anti-inflammatory drugs, and pain relievers for a month before inclusion in the study.

Blood samples were collected in the morning after night fasting (at least 6 hours) from the cubital vein at the same time from 8 to 9 am. All blood samples were delivered to the laboratories under the identification number of the study participant; laboratory staff did not have information about the study participant (the main or control group, gender, age and other data were blinded). For plasma separation, blood was collected in vacuum tubes containing ethylenediaminetetraacetic acid (EDTA, lilac label) or sodium heparin (green label); the obtained samples were centrifuged (30 minutes at 3,000 rpm) for 30 minutes after blood collection. Erythrocyte hemolysate was obtained by 20-fold dilution of erythrocyte mass in 15 mM Tris-HCl Buffer, pH = 8.0 (CAS Number: 1185-53-1). Erythrocytes were preliminarily depleted in leukocytes and platelets by 3-fold repeated addition of 0.9% isotonic sodium chloride solution (1 : 3 ratio) and removal of the supernatant after centrifugation (10 min at 3,000 rpm (centrifuge Z206A HERMLE, Germany).

The Cu-Zn superoxide dismutase (SOD) activity in erythrocyte hemolysate was assessed by the method based on the ability of the enzyme to compete with nitro blue tetrazolium for superoxide anions formed as a result of the interaction of the reduced form of nicotinamide adenine dinucleotide (NADH2) and phenazine metasulfate. The quantitative parameters of the ongoing reaction were determined by measuring the optical density of the reaction mixture at a wavelength of 540 nm in a cuvette with an optical path length of 1 cm against the mixture without NADH2. The result obtained was expressed in active units/g Hb [20].

The quantitative determination of the catalase (CAT) activity in the erythrocyte hemolysate was carried out by the method based on the property of hydrogen peroxide (H_2_O_2_) to degrade under the influence of CAT on H_2_O and O_2_ [21]. The CAT activity was evaluated by the change in the concentration of H_2_O_2_ in the sample in 20 seconds with a spectrophotometric method at a wavelength of 240 nm with the maximum absorption of H_2_O_2_, compared to the control (phosphate buffer) in a cuvette with an optical path length of 1 cm. The amount of degraded H_2_O_2_ was determined by the difference in values (for a certain time interval); it corresponds to the CAT activity. The result obtained was expressed in active units/gHb [22].

The GSH content was assessed spectrophotometrically on the basis of the ability of the sulfhydryl group of GSH to react with 5,5-dithio-bis-(2-nitrobenzoic) acid (Ellman's reagent); the measurements were performed on an SF-56 spectrophotometer. The result was expressed in *μ*mol/l [23].

Determination of malondialdehyde (MDA) concentration in blood plasma was carried out according to the method of Folch et al. [20]. The principle of the method is as follows: in an acidic medium at a high temperature of 90-100°C, MDA reacts with 2 molecules of thiobarbituric acid to form a colored trimethyl complex with an absorption maximum at a wavelength of 532 nm. The MDA concentration was expressed in units of optical density relative to the amount of total lipids (nmol/ml plasma).

The oxidative modification of proteins (OMP) degree was assessed using the R.L. Levine's method [21, 23] based on the determination of the content of carbonyl derivatives in blood plasma resulting from spontaneous oxidation (SO) and metal-catalyzed oxidation (MCO) of amino acid residues in the protein molecule of free radicals (FR) or reactive oxygen species (ROS). SO occurs without catalysts; it shows the level of OMP under normal conditions, in case of MCO iron participates in the reaction, catalyzing the generation of FR and MCO shows the body's reserve-adaptive abilities to resist oxidation. The carbonyl derivatives formed by these reactions can quantitatively react with 2,4-dinitrophenylhydrazine to form aldehyde-2,4-dinitrophenylhydrazones and ketone-2,4-dinitrophenylhydrazones (ADNPH and KDNPH, respectively). The concentration of ADNPH and KDNPH was determined spectrophotometrically at a wavelength of 363 nm and 270 nm in a cuvette with an optical path length of 1 cm in comparison with the control sample. The results obtained were expressed in optical density units/mg of protein/ml.

The determination of folate and cobalamin (B12) levels was carried out by the method of chemiluminescent microparticle immunoassay (CMIA, Architect, Abbott lab.S.A.). Hcy concentration was measured with a Cobas analyzer (Roche Diagnostics) using an enzymatic assay.

All patients included in the study underwent a standardized examination using PANSS; the results of laboratory examination were not known to the investigating physician.

The findings were statistically analyzed using Statistica 6.0. As the distribution of the data differed from the normal (W-test Shapiro-Wilk), Mann–Whitney *U*-test (MWU-test) was used. To assess the qualitative variables the frequency tables (Chi-square statistic with Yates correction, *χ*^2^) was used. Spearman's rank correlation (*ρ*) was used to evaluate the correlations. The data are presented using mean ± standard deviation (*m* ± *σ*), median and interquartile range (Me (Q1, Q3)). The differences between the groups were considered statistically significant at *p* < 0.05.

## 3. Results

Hcy in patients is statistically significantly higher than in healthy subjects (13.65 (11.06, 17.47) versus 11.21 (9.29, 12.96) *μ*mol/l, respectively, *z* = −2.99; *p* = 0.0027/Benjamini-Hohberg adjusted *p* = 0.0041), and this may be due to the statistically significantly lower folate level in patients (3.30 (2.40, 4.30) vs. 4.40 (3.50, 5, 40) *μ*mol/l, respectively, *z* = 3.03; *p* = 0.0024/Benjamini-Hohberg adjusted *p* = 0.0072). In patients, a statistically significant negative correlation was found between the level of Hcy both with the level of folate (*ρ* = −0.38, *p* = 0.0063) and with the level of B12 (*ρ* = −0.36, *p* = 0.0082).

At the same time, patients showed statistically significantly higher rates of OMP (КDNPH and ADNPH), determined during SO, lower CAT activity (Table 1). All other markers of oxidative stress are also more pronounced in patients, but not statistically significant with the available number of observations (Table 1). The level of GSH in patients is lower than in healthy volunteers; however, an increase in the sample size is required to confirm the statistical significance of the difference, which is planned to be done in the future in the framework of this study (probability of type II error *β* = 0.38; required sample size 158, power analysis, Statistica 6.0).

However, according to the results of the correlation analysis, Hcy is not associated with the studied markers of oxidative stress in patients; there was not even a tendency to obtain a significant association in the future, except for a tendency for a negative correlation with the activity of CAT in blood plasma, one of the antioxidant defence enzymes, which is practically not represented in the central nervous system (Table 2).

The possible influence of confounding variables was taken into account (using the method of partial correlation): sex and age, as Hcy level had a correlation with male sex (*ρ* = 0.359, *p* = 0.01), but the analysis showed that the above variables did not significantly distort the relationship between Hcy and GSH. In addition, a possible nonlinear relationship between the studied parameters was worked out, but this assumption was not confirmed.

In the group of patients with an increased level of Hcy (>10 *μ*mol/l, *n* = 42) compared with other patients (*n* = 8), some negative symptoms (PANSS) were statistically significantly more pronounced: difficulty in abstract thinking (N5, *p* = 0.019), lack of spontaneity and flow in conversation (N6, *p* = 0.022), stereotyped thinking (N7, *p* = 0.013), and motor retardation (G7, *p* = 0.050).

## 4. Discussion

Thus, in this study, in patients with schizophrenia, hyperhomocysteinemia caused by deficiency of folate and B12 is confirmed and can be considered a marker of disturbances of vitamin metabolism. This complements the previously obtained data on the genetic background for disturbances of folate metabolism in schizophrenia in the Russian population [24] confirming that hyperhomocysteinemia in patients is associated with disturbances of B vitamins metabolism, as well as the data available in the literature that hyperhomocysteinemia is an integral marker of folate metabolism disturbances in schizophrenia [2, 4]. This is consistent with the data of other authors that the level of Hcy in schizophrenia is higher than in healthy control and is associated with a deficiency of folate and B12 [3]. Moreover, Roffman et al. (2018) previously showed that L-methylfolate supplementation which lowered Hcy levels was associated with salutary physiologic changes and selective symptomatic improvement in schizophrenia patients [25]. This indicates an important pathogenetic role of vitamins (folate and В12) in the development of biochemical disturbances in schizophrenia (including hyperhomocysteinemia). At the same time, whether Hcy has its own pathogenetic activity remains unclear.

According to our data the markers of the redox imbalance are also statistically more pronounced in schizophrenia than in healthy control. But, the redox imbalance is probably not directly related to hyperhomocysteinemia and is hypothetically caused by other pathological processes, as according to the results of the correlation analysis; Hcy is not associated with the studied markers of oxidative stress in patients (even at the trend level, taking into account the analysis of possible confounding factors).

In addition, according to the data obtained, an indirect effect of Hcy on the enzymatic antioxidant defense system (CAT activity) cannot be ruled out, which requires further exploration and can be confirmed later with an increase in the sample size. Given that the ability of Hcy to decrease CAT activity has been found in other chronic neurodegenerative disorders [26], the study of the association of Hcy with CAT activity and the consequences of this association for the redox system in schizophrenia is of particular relevance for understanding the molecular mechanisms of pathogenesis of this disorder. However, in this case Hcy is not a direct prooxidant agent, but it influences the antioxidant enzymatic defence. Thus, the experimentally shown prooxidant activity of high levels of Hcy should not be extrapolated to the *in vivo* situation in schizophrenia.

These preliminary results require further testing a number of hypotheses. One of the ways of Hcy utilization is the transsulfuration pathway: cystathionine-*β*-synthase (CBS) is an enzyme (with vitamin B6 as an essential cofactor) that converts Hcy to cysteine. Cysteine is one of the key determinants of GSH synthesis. GSH is the most important antioxidant of neuronal tissue [13]. So, Hcy can play a protective role under conditions of oxidative stress as a precursor of GSH synthesis. Some experimental studies have already shown that the synthesis of GSH from Hcy is increased under the influence of prooxidants (the transsulfuration pathway is activated), and vice versa, under the influence of antioxidants this pathway is inhibited [19]. These studies indicate the reciprocal sensitivity of the transsulfuration pathway to pro- and antioxidants. The rationale for such regulation may be that an increase in the flow of Hcy through the transsulfuration pathway under conditions of oxidative stress will lead to an increase in GSH synthesis and, thus, will serve as an autocorrecting response to oxidative damage. Under the opposite conditions, Hcy will be redirected mainly to the remethylation pathway leading to its recirculation. So presumably, Hcy metabolism can vary depending on the level of oxidative stress for donating GSH due to redistribution between the associated biochemical processes of transsulfuration and methylation.

In separate cases, an excess of Hcy and simultaneous deficiency of GSH can be the consequences of violation in the transsulfuration pathway (due to a pyridoxine deficiency or a disruption in the functioning of CBS), while Hcy is the only an artefact of an enzyme blockade for which it is a substrate. In support of this hypothesis, there are a number of studies according to which there is a B6 deficiency in schizophrenia, as well as genetic markers of transsulfuration disorders. The manifestation of mental disorders (but not schizophrenia) with CBS deficiency has been described for a long time [27], and in the Russian population, data were obtained on the association of the 844ins68 CBS genetic polymorphism with schizophrenia [28]. In a recently published review, the authors draw attention to the problem of the transsulfuration pathway in treatment-resistant schizophrenia [29]. However, our data suggest the presence of these disorders not only in patients with treatment-resistant schizophrenia. Among the patients of our sample in 21 out of 50 cases hyperhomocysteinemia (above 10 *μ*mol/l) is combined with GSH levels below the reference values (950-1250 *μ*mol/l), which may indirectly indicate a violation of transsulfuration. This suggests the need to study the activity of CBS (genetic polymorphisms of the enzyme, deficiency of its cofactor B6, and the amino acid composition of serum) at least in this subgroup of patients. Considering the large proportion of this subgroup in the studied sample (42%), the study of this metabolic pathway is relevant among all patients with schizophrenia. As part of this work, in the future, it is planned to genotype DNA samples of all participants for genetic polymorphisms that affect the transsulfuration of Hcy.

The limitations of this study were the small sample size (data collection is ongoing), the lack of an assessment of the participants' diet, smoking, lack of serum amino acid assessment, and lack of assessment of genetic polymorphisms affecting Hcy metabolism.

Further study of the role of Hcy in the pathogenesis of schizophrenia is relevant, since the proportion of patients with hyperhomocysteinemia is high and correlations of its level with negative symptoms of schizophrenia are noted, which does not exclude the possibility of its contribution to the prognosis of the disease.

## Figures and Tables

**Table 1:** Markers of oxidative stress in patient and healthy control groups.

Parameter	Patients (*n* = 50)	Healthy (*n* = 36)	Mann–Whitney test; *p*/Benjamini-Hohberg adjusted *p*
GSH, *μ*Mol/l: *m* ± *σ*, Me (Q1, Q3)	935.5 ± 193.5; 923.5 (798, 1047)	998.7 ± 136.0; 1002 (898.5, 1075.5)	*Z* = −1.94; *p* = 0.052/*p* = 0.10
SOD activity, units of activity/g Hb: *m* ± *σ*, Me (Q1, Q3)	197.3 ± 124.1; 164.5 (137, 219)	198.2 ± 88.8; 180 (138.5, 223)	*z* = −0.74; *p* = 0.46/*p* = 0.53
CAT activity, units of activity/g Hb: *m* ± *σ*, Me (Q1, Q3)	44.6 ± 33.7; 41.5 (17, 70)	68.6 ± 35.93; 60.0 (40.5, 93.0)	*z* = −2.92; *p* = 0.0035/*p* = 0.014
КDNPH, spontaneous oxidation (units optical density/mg protein/ml): *m* ± *σ*, Me (Q1, Q3)	0.11 ± 0.023; 0.11 (0.10, 0.13)	0.093 ± 0.016; 0.090 (0.08, 0.10)	*z* = 4.02; *p* = 0.000058/*p* = 0.00046
ADNPH, spontaneous oxidation (units optical density/mg protein/ml): *m* ± *σ*, Me (Q1, Q3)	0.082 ± 0.024; 0.080 (0.060, 0.090)	0.067 ± 0.024, 0.070 (0.050, 0.080)	*z* = 2.44; *p* = 0.014/*p* = 0.037
КDNPH, metal-catalyzed oxidation (units optical density/mg protein/ml): *m* ± *σ*, Me (Q1, Q3)	0.50 ± 0.067; 0.49 (0.45, 0.53)	0.49 ± 0.074; 0.48 (0.44, 0.53)	*z* = 0.35; *p* = 0.73/*p* = 0.73
АDNPH, metal-catalyzed oxidation (units optical density/mg protein/ml): *m* ± *σ*, Me (Q1, Q3)	0.48 ± 0.077; 0.46 (0.43, 0.51)	0.46 ± 0.10; 0.44 (0.39, 0.53)	*z* = 1.04; *p* = 0.30/*p* = 0.48
MDA, nmol/ml: *m* ± *σ*, Me (Q1, Q3)	3.45 ± 0.98; 3.15 (2.92, 4.05)	3.16 ± 0.74; 3.21 (2.65, 3.71)	*z* = 0.96; *p* = 0.33/*p* = 0.44

Note: GSH: reduced glutathione; SOD: superoxide dismutase; CAT: catalase; КDNPH: ketone-2,4-dinitrophenylhydrazones; АDNPH: aldehyde-2,4-dinitrophenylhydrazones; MDA: malonic dialdehyde; Me (Q1, Q3): median and interquartile range; “*m* ± *σ*”: mean ± standard deviation.

**Table 2:** Spearman's correlation coefficient of Hcy with markers of oxidative stress in the studied samples.

	Hcy patients		Hcy healthy	
	*ρ*	*p* level	*ρ*	*p* level
GSH (*μ*Mol/l)	0.098	0.498	-0.263	0.120
SOD activity (units of activity/g Hb)	0.055	0.703	0.209	0.221
CAT activity (units of activity/g Hb)	-0.252	0.078	-0.0046	0.979
КDNPH, spontaneous oxidation (units optical density/mg protein/ml)	-0.085	0.557	-0.224	0.189
ADNPH, spontaneous oxidation (units optical density/mg protein/ml)	-0.069	0.634	0.073	0.670
КDNPH, metal-catalyzed oxidation (units optical density/mg protein/ml)	-0.050	0.730	-0.078	0.652
АDNPH, metal-catalyzed oxidation (units optical density/mg protein/ml)	-0.088	0.543	0.037	0.830
MDA (nmol/ml)	-0.150	0.298	0.381	0.022

Note: Hcy: homocysteine; GSH: reduced glutathione; SOD: superoxide dismutase; CAT: catalase; КDNPH: ketone-2,4-dinitrophenylhydrazones; АDNPH: aldehyde-2,4-dinitrophenylhydrazones; MDA: malonic dialdehyde; Me (Q1, Q3): median and interquartile range; “*m* ± *σ*”: mean ± standard deviation; *ρ*: Spearman's correlation coefficient.

## Data Availability

Data is available on request: bizet@inbox.ru
